# Cardiovascular disease and mortality sequelae of COVID-19 in the UK Biobank

**DOI:** 10.1136/heartjnl-2022-321492

**Published:** 2022-10-24

**Authors:** Zahra Raisi-Estabragh, Jackie Cooper, Ahmed Salih, Betty Raman, Aaron Mark Lee, Stefan Neubauer, Nicholas C. Harvey, Steffen E. Petersen

**Affiliations:** 1 William Harvey Research Institute, NIHR Barts Biomedical Research Centre, Queen Mary University of London, London, UK; 2 Barts Heart Centre, St Bartholomew’s Hospital, Barts Health NHS Trust, London, UK; 3 Division of Cardiovascular Medicine, Radcliffe Department of Medicine, National Institute for Health Research Oxford Biomedical Research Centre, University of Oxford, Oxford, UK; 4 MRC Lifecourse Epidemiology Centre, University of Southampton, Southampton, UK; 5 NIHR Southampton Biomedical Research Centre, University of Southampton and University Hospital Southampton NHS Foundation Trust, Southampton, UK; 6 Health Data Research UK, London, UK; 7 Alan Turing Institute, London, UK

**Keywords:** COVID-19, epidemiology

## Abstract

**Objective:**

To examine association of COVID-19 with incident cardiovascular events in 17 871 UK Biobank cases between March 2020 and 2021.

**Methods:**

COVID-19 cases were defined using health record linkage. Each case was propensity score-matched to two uninfected controls on age, sex, deprivation, body mass index, ethnicity, diabetes, prevalent ischaemic heart disease (IHD), smoking, hypertension and high cholesterol. We included the following incident outcomes: myocardial infarction, stroke, heart failure, atrial fibrillation, venous thromboembolism (VTE), pericarditis, all-cause death, cardiovascular death, IHD death. Cox proportional hazards regression was used to estimate associations of COVID-19 with each outcome over an average of 141 days (range 32–395) of prospective follow-up.

**Results:**

Non-hospitalised cases (n=14 304) had increased risk of incident VTE (HR 2.74 (95% CI 1.38 to 5.45), p=0.004) and death (HR 10.23 (95% CI 7.63 to 13.70), p<0.0001). Individuals with primary COVID-19 hospitalisation (n=2701) had increased risk of all outcomes considered. The largest effect sizes were with VTE (HR 27.6 (95% CI 14.5 to 52.3); p<0.0001), heart failure (HR 21.6 (95% CI 10.9 to 42.9); p<0.0001) and stroke (HR 17.5 (95% CI 5.26 to 57.9); p<0.0001). Those hospitalised with COVID-19 as a secondary diagnosis (n=866) had similarly increased cardiovascular risk. The associated risks were greatest in the first 30 days after infection but remained higher than controls even after this period.

**Conclusions:**

Individuals hospitalised with COVID-19 have increased risk of incident cardiovascular events across a range of disease and mortality outcomes. The risk of most events is highest in the early postinfection period. Individuals not requiring hospitalisation have increased risk of VTE, but not of other cardiovascular-specific outcomes.

**WHAT IS ALREADY KNOWN ON THIS TOPIC**

- Emerging evidence suggests that people with previous COVID-19 have higher risk of subsequent adverse cardiovascular outcomes; however, these studies are mostly retrospective, include only a limited selection of outcomes and do not consider variation of risk by severity of COVID-19.

**WHAT THIS STUDY ADDS**

- In this prospective analysis of 17 871 UK Biobank participants, we demonstrate association of past COVID-19 with increased incidence of a wide range of cardiovascular disease and mortality events.
- These risks were almost entirely confined to those requiring hospitalisation and were highest in the first 30 days postinfection but remained augmented for a prolonged period thereafter.

**HOW THIS STUDY MIGHT AFFECT RESEARCH, PRACTICE OR POLICY**

- Greater attention to management of cardiovascular risk and low threshold for investigations of patients with past COVID-19 hospitalisation are important in prevention and timely treatment of cardiovascular events.
- Further research is required to delineate the period over which the augmented cardiovascular risk following COVID-19 persists.
- Incidence of venous thromboembolism is across all severities of COVID-19 exposure.
- Future studies are needed to address whether specific interventions are needed to mitigate the risk of VTE associated with COVID-19.

## Introduction

COVID-19 has emerged as a major cause of morbidity and mortality worldwide. Several studies have linked exposure to COVID-19 with higher risk of adverse cardiovascular outcomes, even after recovery from the acute illness.^1–3^ Given the high population exposure to COVID-19, these reports may herald a significant imminent public health problem.

There is urgent need to better understand the long-term cardiovascular consequences of COVID-19. However, existing evidence is mostly limited to retrospective studies, includes only a narrow selection of cardiovascular outcomes and lacks adequate consideration of differential risk by COVID-19 severity.^1 2^ It is important to understand whether the augmented cardiovascular risk associated with COVID-19 is limited to those with severe disease or extends to the wider population of individuals with mild manifestations. This information would define the magnitude of any potential public health impact and guide appropriate targeting of healthcare strategies.

We examined associations of COVID-19 exposure with incident cardiovascular disease (CVD) and mortality outcomes in 17 871 UK Biobank cases, independent of shared risk factors and considered differential relationships by severity of COVID-19.

## Methods

### Study population

The UK Biobank includes half a million participants recruited between 2006 and 2010. Individuals aged 40–69 years were identified from National Health Service registers and invited to participate. All participants completed a detailed baseline assessment.^4^ Linkages have been established, for the whole cohort, with Hospital Episode Statistics (HES), primary care records and death registration data. Furthermore, linkages with Public Health England laboratories permit identification of COVID-19 PCR test results.^5^

### Statistical analysis

COVID-19 exposure was defined using disease codes in primary care or HES records, a positive antibody test or a positive PCR test (online supplemental table 1), aligned with UK Biobank recommendations.^6^ The first record of COVID-19 in any of the linked sources was assigned the index time. We excluded cases with <30 days follow-up from the matched analysis. Each COVID-19-infected participant was propensity score-matched to two controls with no record of COVID-19, using nearest neighbours matching and with the date of COVID-19 infection being the index date for the matched controls. The following propensity score variables were included: age, sex, Townsend score (deprivation), body mass index, ethnicity, diabetes, prevalent ischaemic heart disease (IHD), smoking, hypertension, high cholesterol. As the COVID-19 exposure variable is time-dependent, propensity scores were calculated using parameter estimates from a Cox regression model. After matching, all covariates had a standardised difference of <0.012 indicating good balance between the exposed and unexposed groups.

10.1136/heartjnl-2022-321492.supp1 Supplementary data

We included the following incident CVD outcomes identified from HES and death registration data (online supplemental table 2): myocardial infarction (MI), stroke, heart failure, atrial fibrillation (AF), venous thromboembolism (VTE), pericarditis, all-cause mortality, CVD mortality, IHD mortality. Participants with record of the outcome of interest at the index time were excluded from the analysis for that outcome.

Participants were followed until the first episode of a specific outcome, death or the end of follow-up. The censor date was 30 March 2021 giving, on average, 141 days (range 32–395) of prospective follow-up. The study includes the two first waves of COVID-19 in the UK. Details of the viral variants occurring during this period are reported elsewhere.^7^ Vaccination was introduced from December 2020 onwards.

Cox proportional hazard regression was used to estimate association of COVID-19 with incident cardiovascular outcomes reporting HR, 95% CIs and p values. We report associations for the whole sample and by hospitalisation status (non-hospitalised, primary hospital diagnosis, secondary hospital diagnosis). For end points other than all-cause mortality, we looked at cause-specific hazard with death from other causes considered as a competing risk. We accounted for matching by fitting a model stratified on the matched case-control set.^8^

We assessed whether the cardiovascular associations of COVID-19 exposure reduced over time by splitting the data at failure times and fitting an interaction between COVID-19 status and time at risk. Where there was evidence of a time interaction, effects were calculated for events within and after 30 days of infection. We examined associations for the whole sample and by hospitalisation status (none, primary, secondary).

We also conducted sensitivity analysis based on the whole UK Biobank population, with COVID-19 exposure fitted as a time-varying covariate in Cox models (online supplemental figure 1). Variables used in the propensity matching were fitted as covariates. COVID-19 status was assessed by fitting a binary variable and a categorical variable with four levels (unexposed, non-hospitalised, primary hospital diagnosis, secondary hospital diagnosis). Interaction between COVID-19 status and time at risk was used to assess changes in effect sizes over the course of the pandemic. Where significant interaction was found, we calculated effects in the first and second halves of the analysis period. To consider possible selection bias in our hospitalisation variable, we conducted further sensitivity analyses treating hospitalisation as a time-dependent variable so that CVD events occurring before or on the day of admission would be treated as non-hospitalised for COVID-19.

## Results

### Baseline characteristics

**Figure 1:**
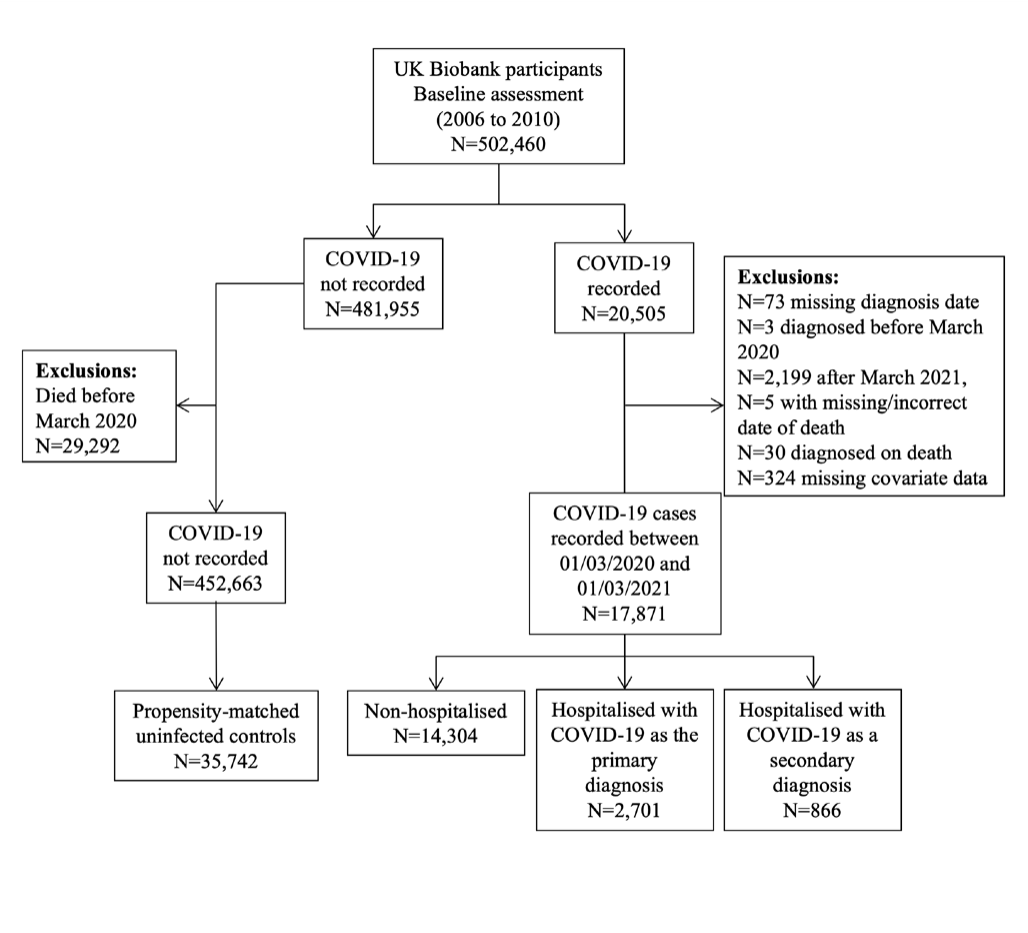
Flow chart of participant selection.

We identified 20 505 participants with record of COVID-19 infection (figure 1). Of these, 17 871 were successfully matched to 35 742 uninfected controls. Compared with the whole UK Biobank cohort, the cases included slightly more men, were less affluent and had poorer cardiometabolic health (table 1). After matching, the cases and controls were well-balanced on these variables.

**Table 1:** Baseline* participant characteristics

	All n=471 227	Whole UK Biobank		Matched cohort	
		COVID-19 n=18 564	Non-COVID-19 n=452 663	COVID-19 cases n=17 871	Matched controls n=35 742
Men, n (%)	210 730 (44.7%)	8743 (47.1%)	201 987 (44.6%)	8409 (47%)	16 813 (47%)
Women, n (%)	260 496 (55.3%)	9821 (52.9%)	250 675 (55.4%)	9462 (53%)	18 929 (53%)
Age on 1 March 2020, median (IQR)	69 (62, 75)	65 (58, 73)	69 (62, 75)	65 (58, 73)	65 (58, 73)
BMI, median (IQR)	26.7 (24.1, 29.9)	27.6 (24.8, 30.9)	26.7 (24.1, 29.8)	27.5 (24.8, 30.9)	27.4 (24.6, 30.9)
BAME	25 875 (5.5%)	1838 (10.0%)	24 037 (5.3%)	1752 (9.8%)	3381 (9.5%)
Diabetes	34 039 (7.2%)	1920 (10.3%)	32 119 (7.1%)	1835 (10.3%)	3683 (10.3%)
Prevalent IHD	48 452 (10.3%)	2275 (12.3%)	46 177 (10.2%)	2184 (12.2%)	4398 (12.3%)
Prevalent AF	26 633 (5.7%)	1273 (6.9%)	25 360 (5.6%)	1420 (8.0%)	1911 (5.4%)
Prevalent HF	10 260 (2.2%)	662 (3.6%)	9598 (2.1%)	808 (4.5%)	856 (2.4%)
Current smoking	46 994 (10.0%)	2162 (11.7%)	44 832 (9.9%)	2096 (11.7%)	4172 (11.7%)
Hypertension	157 538 (33.4%)	6502 (35.0%)	151 036 (33.4%)	6241 (34.9%)	12 474 (34.9%)
High cholesterol	83 595 (17.7%)	3475 (18.7%)	80 120 (17.7%)	3336 (18.7%)	6689 (18.7%)
Townsend score, median (IQR)	−2.17 (−3.66, 0.49)	−1.46 (−3.27, 1.63)	−2.19 (−3.67, 0.43)	−1.48 (−3.27, 1.61)	−1.50 (−3.32, 1.58)

*Age, diabetes, prevalent IHD and hypertension in March 2020.

AF, atrial fibrillation; BAME, black Asian and minority ethnic; BMI, body mass index; CVD, cardiovascular disease; HF, heart failure; IHD, ischaemic heart disease.;

Most participants with past COVID-19 were not hospitalised (80.0%, n=14 304). Among the 20.0% (3567/17 871) of cases recorded in hospital, 75.7% (2701/3567) were primary COVID-19 hospitalisations, while 24.3% (866/3567) had COVID-19 recorded as a secondary diagnosis.

### Observed events

We observed at least one incident event in 9.0% (1616/17 871) of cases and 0.7% (241/35 742) of controls (table 2). Among the cases, 3.0% (534/17 871) developed at least one of the incident CVDs considered, compared with 0.5% (180/35 742) in the controls.

**Table 2:** Numbers and incident rates by COVID-19 status for each model

		All case-control sets	Cases with no hospital admission record	Cases hospitalised with primary COVID-19 diagnosis	Cases hospitalised with secondary COVID-19 diagnosis
MI					
Controls	Number of events Rate/1000 py	35 2.66	30 3.08	4 1.51	1 3.66
Cases	Number of events Rate/1000 py	31 5.19	3 0.62	18 20.81	10 34.55
Stroke					
Controls	Number of events Rate/1000 py	24 1.79	16 1.61	3 1.11	5 6.32
Cases	Number of events Rate/1000 py	48 8.02	14 2.90	24 27.09	10 35.85
Heart failure					
Controls	Number of events Rate/1000 py	47 3.49	33 3.31	9 3.34	5 6.21
Cases	Number of events Rate/1000 py	126 21.02	14 2.87	85 99.40	27 98.87
AF					
Controls	Number of events Rate/1000 py	69 5.28	47 4.86	20 7.66	2 2.57
Cases	Number of events Rate/1000 py	172 29.91	24 5.08	125 160.63	23 92.74
VTE					
Controls	Number of events Rate/1000 py	26 1.95	14 1.41	10 3.76	2 2.51
Cases	Number of events Rate/1000 py	166 27.84	19 3.94	126 147.74	21 70.70
Pericarditis					
Controls	Number of events Rate/1000 py	6 0.43	3 0.29	3 1.09	0 0.0
Cases	Number of events Rate/1000 py	24 3.84	1 0.20	20 20.95	3 9.28
All-cause death					
Controls	Number of events Rate/1000 py	85 7.9	60 7.7	19 8.23	6 8.9
Cases	Number of events Rate/1000 py	1296 265.4	266 70.16	832 1022.31	198 712.12
CVD death					
Controls	Number of events Rate/1000 py	19 1.76	13 1.67	3 1.29	3 4.45
Cases	Number of events Rate/1000 py	48 9.83	13 3.42	13 15.97	22 79.21
IHD death					
Controls	Number of events Rate/1000 py	12 1.11	10 1.29	1 0.43	1 1.48
Cases	Number of events Rate/1000 py	24 4.92	5 1.31	7 8.60	12 43.15

AF, atrial fibrillation; CVD, cardiovascular disease; IHD, ischaemic heart disease; MI, myocardial infarction; py, person-years; VTE, venous thromboembolism.

Incident CVDs occurred markedly more commonly in hospitalised cases than in controls. The top three most common incident CVDs among the cases were incident AF, VTE and heart failure. The highest rates of these conditions were observed in participants with a record of primary COVID-19 hospitalisation. In those without record of hospitalisation, there were less marked differences in rates of incident CVDs between cases and controls, but with greater rates of incident stroke, AF and VTE among cases.

A total of 7.3% (1296/17 871) of the cases died compared with 0.2% (85/35 742) of controls. Those with primary COVID-19 hospitalisation had the highest rates of death (table 2). There were higher rates of CVD and IHD death in cases compared with controls; this was more marked in individuals with a record of hospitalisation. Individuals admitted with COVID-19 as a secondary diagnosis had the highest rates of death due to CVD and IHD (table 2).

### Non-hospitalised participants with COVID-19

**Figure 2:**
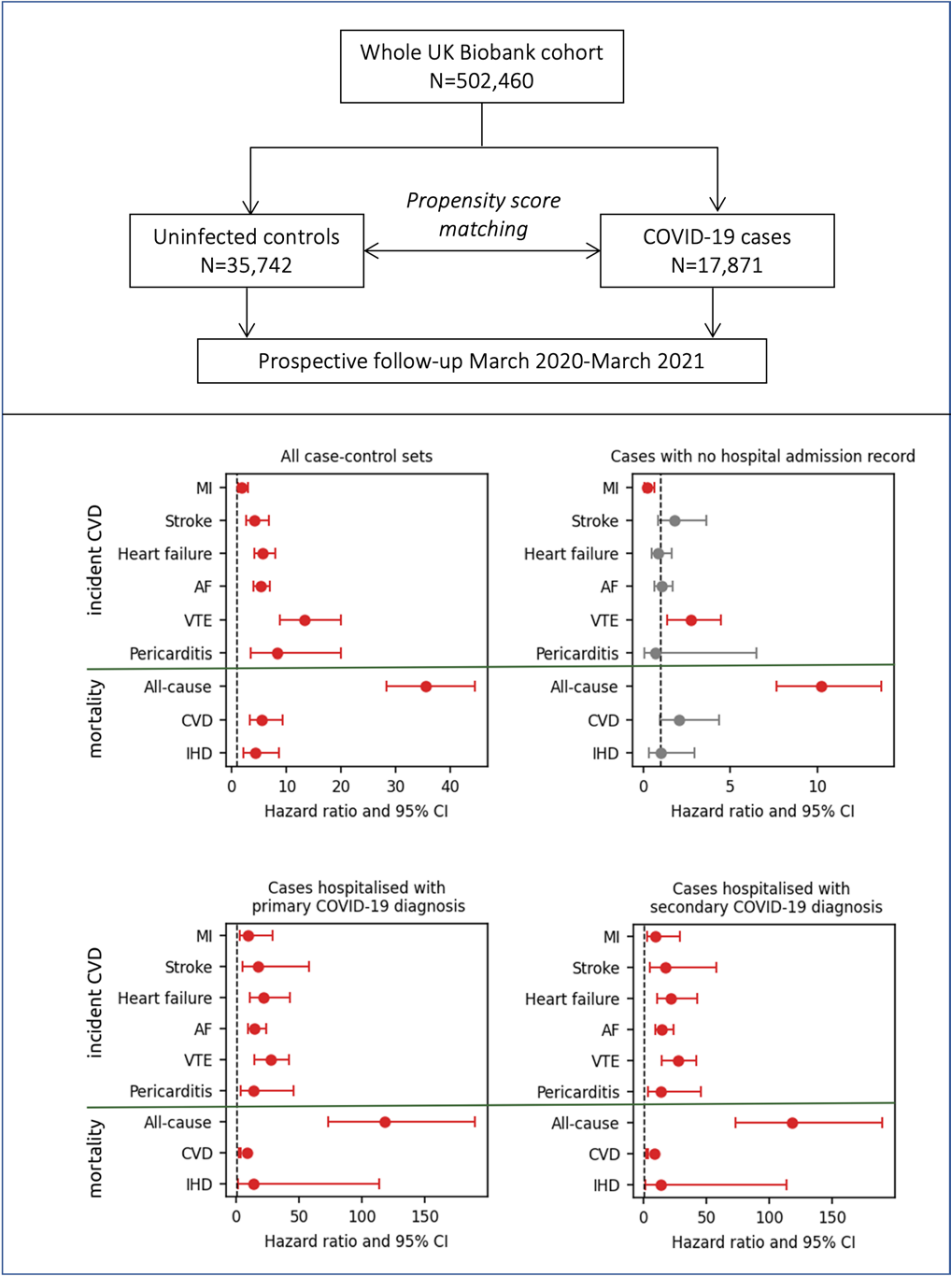
Summary of study design and results. AF, atrial fibrillation; CVD, cardiovascular disease; IHD, ischaemic heart disease; MI, myocardial infarction; VTE, venous thromboembolism. Red bars indicate statistically significant associations (p<0.05).

Compared with matched uninfected controls, non-hospitalised participants with COVID-19 (n=14 304) had over 2.7-fold greater risk of incident VTE (HR 2.7 (95% CI 1.4 to 5.5); p=0.004) and over 10-fold greater risk of all-cause death (HR 10.2 (95% CI 7.6 to 13.7); p<0.0001). There was no significant association with risk of the other outcomes, with the exception of incident MI, which was significantly lower than in controls (table 3, figure 2).

**Table 3:** Associations of COVID-19 case-control status with incident events

	All case-control sets n=17 871*	Cases with no hospital admission record n=14 304	Cases hospitalised with primary COVID-19 diagnosis n=2701	Cases hospitalised with secondary COVID-19 diagnosis n=866
**Incident diseases**	**HR (95% CI)**	**HR (95% CI)**	**HR (95% CI)**	**HR (95% CI)**
MI	1.82 (1.12 to 2.96) P=0.015	0.19 (0.06 to 0.65) P=0.008	9.9 (3.36 to 29.1) P<0.0001	22.2 (2.84 to 173) P=0.003
Stroke	4.15 (2.54 to 6.78) P<0.0001	1.77 (0.86 to 3.63) P=0.12	17.5 (5.26 to 57.9) P<0.0001	4.54 (1.55 to 13.33) P=0.006
Heart failure	5.6 (4.05 to 7.87) P<0.0001	0.85 (0.45 to 1.61) P=0.63	21.6 (10.9 to 42.9) P<0.0001	13.1 (5.06 to 33.8) P<0.0001
AF	5.25 (3.98 to 6.93) P<0.0001	1.03 (0.63 to 1.69) P=0.90	14.9 (9.34 to 23.8) P<0.0001	29.3 (6.94 to 124) P<0.0001
VTE	13.2 (8.75 to 19.9) P<0.0001	2.74 (1.38 to 5.45) P=0.004	27.6 (14.5 to 52.3) P<0.0001	23.1 (5.42 to 98.4) P<0.0001
Pericarditis	8.21 (3.38 to 20.00) P<0.0001	0.68 (0.07 to 6.48) P=0.74	13.6 (4.06 to 45.8) P<0.0001	– –

Mortality	HR (95% CI)	HR (95% CI)	HR (95% CI)	HR (95% CI)
All-cause	35.47 (28.24 to 44.55) P<0.0001	10.23 (7.63 to 13.70) P<0.0001	118.01 (73.32 to 189.95)P<0.0001	63.97 (30.14 to 135.77) P<0.0001
CVD	5.48 (3.24 to 9.29) P<0.0001	2.02 (0.93 to 4.34) P=0.073	8.76 (2.51 to 30.5) P=0.001	14.6 (4.37 to 48.8) P<0.0001
IHD	4.23 (2.16 to 8.67) P<0.0001	1.00 (0.34 to 2.93) P=0.99	14.1 (1.73 to 113.8) P=0.013	23.7 (3.09 to 182) P=0.002

*Sample sizes indicate the number of cases; the analysis sample also includes two controls per case.

AF, atrial fibrillation; CVD, cardiovascular disease; IHD, ischaemic heart disease; MI, myocardial infarction; VTE, venous thromboembolism.

### Participants with primary COVID-19 hospitalisation

Individuals with record of primary COVID-19 hospitalisation (n=2701) had significantly increased risk of all outcomes considered (table 3, figure 2). The largest effect sizes were observed with incident VTE (HR 27.6 (95% CI 14.5 to 52.3); p<0.0001), heart failure (HR 21.6 (95% CI 10.9 to 42.9); p<0.0001) and stroke (HR 17.5 (95% CI 5.3 to 57.9); p<0.0001). The risk of incident AF was increased by almost 15-fold, pericarditis by near 14-fold and MI by almost 10-fold (table 3).

The risk of all-cause death was increased by 118-fold (HR 118.0 (95% CI 73.32 to 190.0); p<0.0001), primary CVD death by near 9-fold (HR 8.8 (95% CI 2.5 to 3.5); p=0.001) and IHD death by over 14-fold (HR 14.1 (95% CI 1.7 to 113.8); p=0.013).

### Participants with secondary COVID-19 hospitalisation

Participants with secondary COVID-19 hospitalisation (n=866) had increased risk of all incident outcomes compared with uninfected controls (table 3). Their risk of all-cause death was less augmented than in those with primary COVID-19 hospitalisation, while their risk of death due to CVD or IHD was higher. Similarly, the risk of incident MI and AF was increased to a greater extent than in those with primary COVID-19 hospitalisation. While the risk of incident heart failure, stroke and VTE were increased compared with controls, the risk of these conditions was less augmented than in those with a primary COVID-19 admission. There were too few pericarditis cases in this subset for assessment of associations. The most common primary reasons for admission in this subset are presented in online supplemental table 3.

### Modification of risk with time

**Figure 3:**
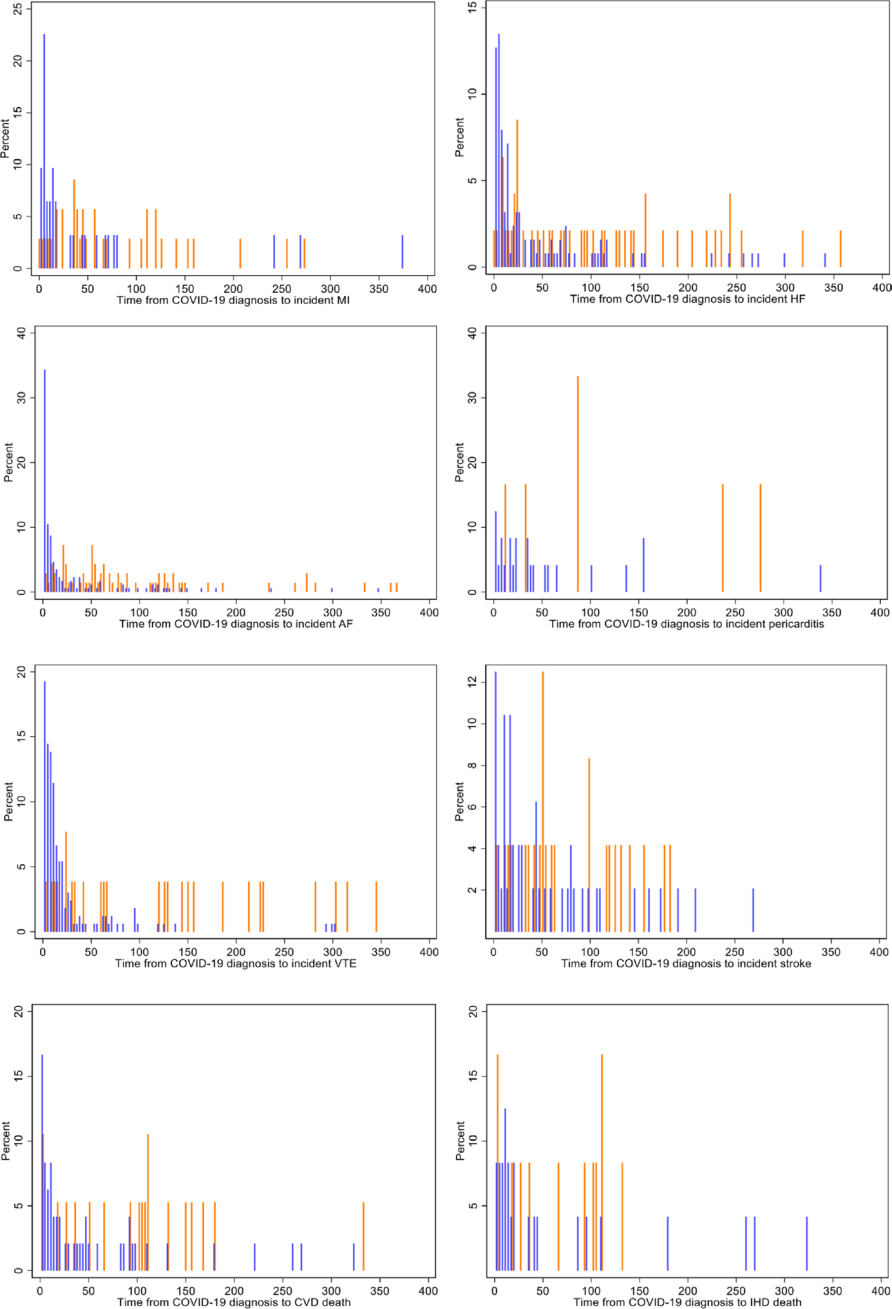
Histograms of event time for all incident outcomes. Blue bars represent incident events in COVID-19 cases, while the red bars indicate those in matched controls. AF, atrial fibrillation; CVD, cardiovascular disease; HF, heart failure; IHD, ischaemic heart disease; MI, myocardial infarction; VTE, venous thromboembolism.

We considered whether the risk of incident events varied with time from COVID-19 diagnosis. Most events occurred in the early postinfection period, typically within 30 days of infection (figure 3). There was evidence of significant interaction with time for heart failure, AF, VTE, pericarditis and all-cause death outcomes (table 4). For these events, we ran separate models stratified by time from COVID-19 status (within 30 days and after 30 days to capture the early postinfection period). The increased risk of these outcomes in the cases remained statistically significant across both time strata, but with smaller effect sizes beyond the initial 30-day period (table 4).

**Table 4:** Interactions with time since COVID-19 diagnosis

Incident disease	Interaction with time*	Events within 30 days	Events after 30 days
	Interaction HR (95% CI)	HR (95% CI)	HR (95% CI)
MI	0.996 (0.989 to 1.003) P=0.30	–	–
Stroke	0.992 (0.984 to 1.001) P=0.07	–	–
Heart failure	0.992 (0.988 to 0.997) P=0.001	11.03 (5.97 to 20.38) P<0.0001	2.78 (1.71 to 4.51) P<0.0001
AF	0.986 (0.981 to 0.992) P<0.0001	15.45 (8.87 to 26.91) P<0.0001	1.72 (1.11 to 2.67) P=0.015
VTE	0.984 (0.977 to 0.992) P<0.0001	66.78 (24.7 to 180.5) P<0.0001	3.97 (2.10 to 7.53) P<0.0001
Pericarditis	0.989 (0.979 to 0.999) P=0.04	24.74 (3.22 to 189.9) P=0.002	4.64 (1.63 to 13.19) P=0.004
Mortality			
All-cause	0.020 (0.007 to 0.056) P<0.0001	101.1 (64.9 to 157.3) P<0.0001	9.73 (7.13 to 13.28) P<0.0001
CVD	0.994 (0.987 to 1.001) P=0.12	–	–
IHD	1.00 (0.992 to 1.009) P=0.945	–	–

*For outcomes with a significant time interaction term, we examine associations stratified by 30 days before/after infection date to examine whether observed risks extend beyond the acute postinfection period.

AF, atrial fibrillation; CVD, cardiovascular disease; IHD, ischaemic heart disease; MI, myocardial infarction; VTE, venous thromboembolism;

### Sensitivity analysis

We ran a sensitivity analysis using the entire UK Biobank cohort (n=471 227 participants) alive on 1 March 2020 with inclusion of COVID-19 as a time-varying exposure (online supplemental figure 1). Overall, the pattern of associations was similar to the matched analysis, but with smaller effects for most outcomes in the primary COVID-19 hospitalisation subset (online supplemental table 4). There was significant interaction with time for all outcomes except MI and IHD death, with effect sizes decreasing with increasing time from March 2020 (online supplemental table 5). When treating hospital admission as a time-dependent variable, the effect of COVID-19 on MI was no longer significant (online supplemental table 6).

## Discussion

### Summary of findings

We studied 17 871 UK Biobank participants with exposure to COVID-19 from 1 March 2020 to 1 March 2021 and 35 742 propensity score-matched uninfected controls, considering the risk of a range of incident CVD and mortality outcomes. Individuals with past COVID-19 exposure had greater risk of incident CVDs and mortality, compared with matched uninfected controls.

Participants who were hospitalised with COVID-19 had increased risk of all incident outcomes considered (MI, stroke, heart failure, AF, VTE, pericarditis, all-cause death, CVD death, IHD death), independent of baseline demographic and cardiometabolic factors. Non-hospitalised participants with COVID-19 had significantly greater risk of incident VTE and all-cause mortality, but not of other outcomes. Cardiovascular risks were greatest in the first 30 days after infection but remained higher than controls even after this period.

### Comparison with existing work

We found significantly increased risk of VTE in both hospitalised and non-hospitalised cases, which remained elevated throughout the entire follow-up period. Our observations are broadly consistent with self-controlled case series analyses from Scotland^9^ and Sweden,^10^ a retrospective cohort study from the USA^2^ and a large prospective cohort study from the USA.^3^ Furthermore, a recent analysis of almost 1 million COVID-19 cases across four European nations demonstrated venous and arterial thrombosis in both hospitalised and non-hospitalised cases.^11^

Currently, the National Institute of Health and Care Excellence recommends prophylactic low molecular weight heparin for VTE prevention in hospitalised patients with COVID-19 and in patients who would otherwise be admitted to hospital (eg, hospital at home) for a minimum of 7 days.^12^ These recommendations are consistent with those of the British Thoracic Society^13^ and the American Society of Hematology.^14^ Our results indicate that the risk of VTE is also increased in non-hospitalised individuals. Overall, available evidence supports a distinct mechanistic role for COVID-19 in driving higher VTE rates which occurs across disease severities and extends beyond the early postinfection phase.

In our study, non-hospitalised individuals with mild COVID-19 had increased risk of VTE, but not of any other cardiovascular outcome. In contrast, a recent prospective analysis of US data by Xie *et al* 3 reports increased risk of a range of cardiovascular outcomes across all disease severities. There are important differences in baseline health status (eg, obesity) and healthcare systems of the UK and the US populations, which may influence both occurrence and recording of cardiovascular outcomes. Furthermore, the UK Biobank cohort is on average healthier than the general UK population,^15^ which may protect against increased cardiovascular risk from mild COVID-19. Another possibility is that barriers to healthcare access in the US population have led to delays in seeking medical attention for non-acute cardiac symptoms (eg, stable angina) leading to greater risk of presentation with acute events (eg, acute MI). These observations underscore the need for evaluation of long-term cardiovascular risk in individuals with mild COVID-19 across independent cohorts and for assessment of factors which may modify disease susceptibility.

We observed increased risk of incident MI and stroke in participants hospitalised with COVID-19. These observations are consistent with retrospective analysis of a cohort from Sweden,^1^ and prospective analyses from Denmark^16^ and the USA.^3^ In a retrospective cohort study, Merkler *et al* 17 demonstrate increased risk of ischaemic stroke in patients hospitalised with COVID-19 compared to those with influenza, indicating a distinct association between COVID-19 and this outcome. The potential underlying mechanisms include vascular cell involvement, coagulopathy and cytokine-mediated plaque destabilisation.^18^ We additionally observed increased rates of incident AF, heart failure and pericarditis among hospitalised COVID-19 cases. There is little data on these outcomes in existing work, but our findings are broadly in keeping with available research.^2 3^

In our main analysis, we found an unexpected association of COVID-19 with lower risk of incident MI in the non-hospitalised subset. It is likely that this finding is a result of selection bias. Individuals who develop mild COVID-19 in the community, but have an MI very soon after would be admitted to hospital and have COVID-19 recorded as a secondary diagnosis. This means that within the non-hospitalised cases we only count events that occur sufficiently separate from the onset of infection, for COVID-19 to not be recorded as a hospital diagnosis. Whereas for their controls, we count events occurring at any time. In effect, the controls have greater time at risk. Indeed, sensitivity analysis using hospitalisation as a time-dependent variable did not show a significant effect of COVID-19 on MI before hospitalisation. In this analysis, we classified individuals whose CVD event was before or on the day of hospitalisation as non-hospitalised, while events after the day of admission was treated as hospitalised. Future studies should be alert to such potential sources of bias, which may produce spurious associations.

Our study is the first to prospectively examine risk of incident primary cardiovascular death in the setting of COVID-19. We observed increased risk of CVD death and IHD death in participants hospitalised with COVID-19. Notably, while the risk of CVD and IHD mortality was significantly elevated in participants with primary COVID-19 hospitalisation, these events were also markedly greater in individuals with COVID-19 as a secondary diagnosis, which likely relate to their primary admission indication rather than COVID-19.

The long-term sequelae of past COVID-19 exposure is emerging as a dominant public health concern. Our findings highlight the increased cardiovascular risk of individuals with past infection, which are likely to be greater in countries with limited access to vaccination and thus greater population exposure to COVID-19. Furthermore, the long-term cardiovascular consequences reported in our study may be relevant in the context of future pandemics of similar viral infections.

### Clinical implications

Our findings indicate increased risk of cardiovascular outcomes following COVID-19, particularly in those requiring hospitalisation. Although most events occur in the early postinfection period, the risk remains augmented for a prolonged period thereafter. Greater attention to management of cardiovascular risk and low threshold for cardiovascular investigations of patients exposed to COVID-19 are important in prevention and timely treatment of cardiovascular events. Further research is required to delineate the period over which the augmented cardiovascular risk persists. Furthermore, more granular analysis of factors which may alter CVD susceptibility following COVID-19 are warranted. The high incidence of VTE in both hospitalised and non-hospitalised cohorts is also concerning. Numerous clinical trials^19 20^ are currently underway to evaluate the role of prolonged prophylactic anticoagulation in patients post-COVID-19 and should provide some long-awaited answers on the benefits of prolonged anticoagulation in this population.

### Strengths and limitations

The large well-characterised sample available through the UK Biobank and extensive health record linkages permitted reliable identification of COVID-19 cases and incident events and creation of a well-balanced matched comparator cohort. We cannot exclude residual confounding from comorbidities not considered in our matching approach (eg, renal disease, cancer). However, given the low prevalence of such factors in the UK Biobank, their omission is unlikely to substantially influence the observed associations. Furthermore, we did not consider the influence of cardiovascular medications, such as statins or ACE inhibitors. Given the significant healthy participant effect in the UK Biobank,^15^ it is possible that our sample was relatively protected from adverse cardiovascular outcomes and this may have resulted in underestimation of risk. Our analysis also highlights the potential for collider bias in COVID-19 studies, which, by nature, select on testing or hospitalisation. It is important that future researchers are alert to such potential sources of bias and undertake dedicated analyses to evaluate and mitigate such factors. We observed significant time-varying nature of risk in our analysis; it is possible that risk of cardiovascular events is further reduced with longer follow-up periods. Our analysis does not consider other potential modifying factors such as the impact of vaccination, new variants of concerns or multiple infection exposures. Such analyses are increasingly relevant as public health approaches to handling of the pandemic evolve.

## Conclusions

In this prospective analysis of 17 871 UK Biobank participants with past COVID-19, we observed increased risk of incident CVD and mortality events in cases compared with uninfected controls, independent of shared demographic and cardiometabolic factors. Overall, our results indicate that while COVID-19 exposure is associated with increased risk of incident adverse cardiovascular events, such risks are almost entirely confined to those with disease requiring hospitalisation and highest in the early (first 30 days) postinfection period.

## Data Availability

Data may be obtained from a third party and are not publicly available. The UK Biobank will make the source data available to all bona fide researchers for all types of health-related research that is in the public interest, without preferential or exclusive access for any persons. All researchers will be subject to the same application process and approval criteria as specified by UK Biobank. For more details on the access procedure, see the UK Biobank website: http://www.ukbiobank.ac.uk/register-apply http://www.ukbiobank.ac.uk/register-apply.
