# Supplementary material for: Cardiovascular disease and mortality sequelae of COVID-19 in the UK Biobank
Source: Heart. 2022 Oct 24;109(2):119–26. doi: 10.1136/heartjnl-2022-321492 (PMC9811071; doi:10.1136/heartjnl-2022-321492)

Supplementary Table 1. Approach to ascertainment of COVID-19 status

Source	Code type	Code details
Primary care	CTV3 codes in TPP	XaLTE<br>Y20d1<br>Y213a<br>Y228d<br>Y22b8<br>Y23f7
	SNOMED-CT codes in EMIS	1240581000000104<br>1300721000000109<br>1321541000000108<br>1321551000000106<br>1321661000000108
	EMIS local code	EMISNQCO303
Public Health England Laboratory data	PCR SARS-CoV-2 test result	Positive
Hospital Episode Statistics	ICD-10	U07.1
Antibody test	SARS-CoV-2 antibody test result	Positive

Supplementary Table 1 footnote. CTV3: Clinical Terms Version 3; EMIS: Egton Medical Information Systems; PCR: polymerase chain reaction; SARS-CoV-2: severe acute respiratory syndrome coronavirus 2; TPP: The Phoenix Partnership.

Supplementary Table 2. Ascertainment of CVD outcomes (ICD and UK Biobank field codes)

Source	ICD code/UKB filed	Description
**Ischaemic heart disease (IHD)**		
ICD9	4139	Angina pectoris
	4140	Coronary atherosclerosis
	4141	Aneurysm of heart
	4148	Other specified forms of chronic ischaemic heart disease
	4149	Chronic ischaemic heart disease, unspecified
	4119	Other acute and subacute forms of ischaemic heart disease
Self-report	20002	Angina
ICD10	I20	Angina pectoris
	I24	Other acute ischaemic heart diseases
	I25	Chronic ischaemic heart disease
First occurrences	131296	Angina pectoris
	131304	Other acute ischaemic heart diseases
	131306	Chronic ischaemic heart disease
Diagnosed by doctor	3627	Age angina diagnosed
	6150: 2	Angina
**Ischaemic heart disease (Myocardial infarction)**		
ICD9	4109	Acute myocardial infarction
Self-report	20002	Heart attack/myocardial infarction
ICD9	410	Acute myocardial infarction
	411	Other acute and subacute forms of ischaemic heart disease
ICD10	I21	Acute myocardial infarction
	I22	Subsequent myocardial infarction
	I23	Certain current complications following acute myocardial infarction
First occurrences	131298	Acute myocardial infarction
	131300	Subsequent myocardial infarction
	131302	Certain current complications following acute myocardial infarction
Diagnosed by doctor	3894	Age heart attack diagnosed
	6150: 1	Heart attack
Algorithm	42000	Date of myocardial infarction
**Heart failure (unspecified aetiology)**		
ICD9	4280	Congestive heart failure
	4281	Left heart failure
Self-report	20002	Heart failure/pulmonary oedema
ICD10	I50.0	Congestive heart failure
	I50.1	Left ventricular failure
	I50.9	Heart failure, unspecified
First occurrences	131354	Heart failure
**Cardiac arrhythmia (Atrial fibrillation)**		
Self-report	20002	Atrial fibrillation
ICD9	4273	Atrial fibrillation and flutter
ICD10	I48.0	Paroxysmal atrial fibrillation
	I48.1	Persistent atrial fibrillation
	I48.2	Chronic atrial fibrillation
	I48.9	Atrial fibrillation and atrial flutter, unspecified
**Stroke**		
Self-report	20002	Stroke
	20002	Ischaemic stroke
	20002	Brain haemorrhage
ICD9	431	Intracerebral haemorrhage
	4349	Occlusion of cerebral arteries, unspecified
ICD10	I64	Stroke, not specified as haemorrhage or infarction
	I63	Cerebral infarction
	I61	Intracerebral haemorrhage
	I62	Other nontraumatic intracranial haemorrhage
First occurrences	131368	Date I64 first reported (stroke, not specified as haemorrhage or infarction)

Source	ICD code/UKB filed	Description
	131366	Cerebral infarction
	131362	Intracerebral haemorrhage
	131364	other nontraumatic intracranial haemorrhage
Diagnosed by doctor	4056	Age stroke diagnosed
	6150: 3	Stroke
Algorithm	42006	Date of stroke
	42008	Date of ischaemic stroke
	42010	Date of intracerebral haemorrhage
**Pericarditis**		
ICD10	I30.0	Acute nonspecific idiopathic pericarditis
	I30.1	Infective pericarditis
	I30.8	Other forms of acute pericarditis
	I30.9	Acute pericarditis, unspecified
	I31.0	Chronic adhesive pericarditis
	I31.1	Chronic constrictive pericarditis
	I31.2	Haemopericardium, not elsewhere classified
	I31.3	Pericardial effusion (noninflammatory)
	I31.8	Other specified diseases of pericardium
	I31.9	Disease of pericardium, unspecified
	I32.0	Pericarditis in bacterial diseases classified elsewhere
	I32.1	Pericarditis in other infectious and parasitic diseases classified elsewhere1
	I32.8	Pericarditis in other diseases classified elsewhere
**Venous thromboembolism (DVT/PE)**		
ICD9	4151	Pulmonary embolism
	4538	Embolism and thrombosis of other specified veins
ICD10	I26.0	Pulmonary embolism with mention of acute cor pulmonale
	I26.9	Pulmonary embolism without mention of acute cor pulmonale
	I801	Phlebitis and thrombophlebitis of femoral vein
	I802	Phlebitis and thrombophlebitis of other deep vessels of lower extremities
	I803	Phlebitis and thrombophlebitis of lower extremities, unspecified
	I82.8	Embolism and thrombosis of other specified veins
	I82.9	Embolism and thrombosis of unspecified vein
Self report	20002	pulmonary embolism +/- DVT
	20002	deep venous thrombosis (DVT)

Supplementary Table 2 footnote. CVD: cardiovascular disease; DVT: deep vein thrombosis; ICD: international classification of disease; PE: pulmonary embolism. For the incident disease outcomes, we considered ICD codes captured via HES or death registration data only. For prevalent disease we included the same ICD codes as well as self-report fields listed above.

Supplementary Table 3. The most common primary reasons for hospitalisation in participants with secondary COVID-19 hospitalisation

ICD-10 code	Primary admission reason	N (%)
A419	Sepsis	25 (2.9%)
I269	Pulmonary embolism	23 (2.7%)
N179	Acute Kidney failure	22 (2.5%)
S7200	Fracture neck of Femur	18 (2.1%)
I500	Heart Failure	15 (1.7%)
J181	Lobar pneumonia	14 (1.6%)
R296	Falls	13 (1.5%)
I639	Cerebral Infarction	12 (1.4%)
N390	Urinary Tract Infection	11 (1.3%)
S7210	Trochanteric Fracture of femur	11 (1.3%)
R55	Syncope	10 (1.2%)

Supplementary Table 3 footnote. COVID-19: coronavirus disease 2019; ICD-10: International Classification of Diseases 10th revision

Supplementary Table 4. Associations of case/control status with incident events- whole cohort analysis

	**All cases N=18564**	**Cases with no hospital admission record N=14,845**	**Cases hospitalised with primary COVID-19 diagnosis N=2,745**	**Cases hospitalised with secondary COVID-19 diagnosis N=974**
**Incident disease**	**HR (95% CI)**	**HR (95% CI)**	**HR (95% CI)**	**HR (95% CI)**
Incident MI	2.01 (1.38, 2.91) P=0.0003	0.30 (0.10, 0.93) P=0.038	4.99 (3,04, 8.20) P<0.0001	7.54 (4.04, 14.10) P<0.0001
Incident stroke	3.53 (2.62, 4.75) P<0.0001	1.60 (0.94, 2.72) P=0.08	7.09 (4.67, 10.76) P<0.0001	7.31 (3.91, 13.65) p<0.0001
Incident HF	4.23 (3.44, 5.20) P<0.0001	0.96 (0.57, 1.63) P=0.88	9.64 (7.42, 12.51) P<0.0001	9.14 (6.14, 13.60) P<0.0001
Incident AF	3.45 (2.85, 4.17) P<0.0001	1.01 (0.67, 1.53) P=0.96	10.41 (8.28, 13.10) P<0.0001	4.32 (2.64, 7.08) P<0.0001
Incident VTE	14.85 (12.39, 17.80) P<0.0001	2.37 (1.50, 3.76) P=0.0002	52.58 (42.74, 64.69) P<0.0001	31.19 (21.30, 45.69) P<0.0001
Incident pericarditis	6.39 (4.04, 10.11) P<0.0001	0.44 (0.06, 3.13) P=0.41	21.35 (12.74, 35.77) P<0.0001	12.75 (4.72, 34.44) P<0.0001
**Mortality outcomes**	**HR (95% CI)**	**HR (95% CI)**	**HR (95% CI)**	**HR (95% CI)**
All-cause	27.6 (25.8, 29.7) P<0.0001	9.58 (8.43, 10.90) P<0.0001	62.7 (57.8, 68.1) P<0.0001	33.15 (28.7, 38.3) p<0.0001
CVD	4.05 (2.96, 5.53) P<0.0001	2.38 (1.39, 4.07) P=0.001	3.61 (2.02, 6.44) P=0.00001	9.99 (6.23, 16.04) P<0.0001
IHD	3.99 (2.65, 6.02) P<0.0001	1.70 (0.76, 3.83) P=0.20	3.12 (1.39, 7.02) P=0.006	15.59 (8.92, 27.27) P<0.0001
Stroke	6.73 (3.81, 11.91) P<0.0001	4.78 (2.07, 11.03) P=0.0003	8.55 (3.44, 21.25) P<0.0001	11.28 (3.91, 32.56) P<0.0001

Supplementary Table 4. Sensitivity analysis is based on 18564 COVID-19 cases and 452,663 unexposed controls. (Total of 20505 cases – exclude 3 before March 2020, 73 with missing diagnosis date, 5 with date of death prior to covid diagnosis. 1860 are diagnosed after censor date and contribute as unexposed only.) AF: atrial fibrillation; CI: confidence interval; COVID-19: coronavirus disease 2019; CVD: cardiovascular disease; HF: heart failure; HR: hazard ratio; IHD: ischaemic heart disease; MI: myocardial infarction; VTE: venous thromboembolism.

Supplementary table 5. Interactions with time since March 2020 in 471,227 participants (cases and controls from UK Biobank)

	**Interaction with time**	**Events in first 6 months**	**Events after 6 months**
**Incident disease in n=471,227 participants**	**Interaction HR (95% CI)**	**HR (95% CI)**	**HR (95% CI)**
MI	0.998 (0.994, 1.002) P=0.398		
Stroke	0.994 (0.991, 0.997) P<0.0001	9.57 (5.71, 16.04) P<0.0001	2.69 (1.88, 3.84) P<0.0001
Heart failure	0.996 (0.994, 0.998) P<0.0001	7.17 (4.68, 10.98) P<0.0001	3.84 (3.05, 4.84) P<0.0001
AF	0.996 (0.994, 0.998) P<0.0001	4.03 (2.42, 6.72) P<0.0001	3.42 (2.80, 4.18) P<0.0001
VTE	0.995 (0.994, 0.997) P<0.0001	28.15 (19.05, 41.61) P<0.0001	12.93 (10.60, 15.79) P<0.0001
pericarditis	0.992 (0.988, 0.997) P=0.001	17.76 (8.64, 36.51) P<0.0001	4.43 (2.49, 7.88) p<0.0001
**Mortality**			
All cause	0.992 (0.991, 0.993) P<0.0001	73.3 (65.9, 81.6) P<0.0001	18.1 (16.6, 19.7) P<0.0001
CVD	0.995 (0.992, 0.998) P=0.002	8.51 (4.65, 15.58) P<0.0001	3.48 (2.45, 4.95) P<0.0001
IHD	0.996 (0.992, 1.000) P=0.083		

Supplementary Table 5 footnote. AF: atrial fibrillation; COVID-19: coronavirus disease 2019; CVD: cardiovascular disease; HR: hazard ratio; IHD: ischaemic heart disease; MI: myocardial infarction; VTE: venous thromboembolism. 6 months is chosen as the midpoint of the study (March 2020-March 2021).

Supplementary Table 6. Associations of case/control status with incident events- whole cohort analysis (alive on 1st March 2020), hospitalisation as a time-dependent variable

	Cases with no hospital admission record N=14,845	Cases hospitalised with primary COVID-19 diagnosis N=2,745	Cases hospitalised with secondary COVID-19 diagnosis N=974
**Incident disease**	**HR (95% CI)**	**HR (95% CI)**	**HR (95% CI)**
Incident MI	1.26 (0.71-2.23) P=0.44	3.51 (1.88-6.56) P<0.0001	2.57 (0.83-7.99) P=0.10
Stroke	1.73 (1.05-2.85) P=0.030	4.42 (2.20-8.88) P<0.0001	5.25 (2.22-12.91) P=0.0002
Heart failure	0.91 (0.50-1.65) P=0.76	6.70 (4.68-9.60) P<0.0001	9.95 (6.38-15.51) P<0.0001
AF	1.14 (0.76-1.71) P=0.53	3.11 (2.00-4.84) P<0.0001	3.48 (1.87-6.59) P<0.0001
VTE	1.74 (1.00-3.02) P=0.050	33.80 (25.67-44.51) P<0.0001	23.39 (14.39-38.0) P<0.0001
Pericarditis	0.92 (0.23-3.72) P=0.91	15.35 (8.08-29.18) P<0.0001	7.32 (1.81-29.54) P=0.005
**Mortality**	**HR (95% CI)**	**HR (95% CI)**	**HR (95% CI)**
All-cause	9.81 (8.66-11.13) P<0.0001	64.22 (59.19) P<0.0001	34.35 (29.73-39.70) P<0.0001
CVD	3.23 (1.36-3.96) P=0.002	3.70 (2.08-6.60) P<0.0001	10.82 (6.79-17.22) P<0.0001
IHD	1.58 (0.70-3.57) P=0.27	2.68 (1.18-6.05) P=0.018	8.21 (4.41-15.28) P<0.0001
Stroke	4.68 (2.03-10.81) P<0.0001	8.87 (3.57-22.04) P<0.0001	11.70 (4.05-33.80) P<0.0001

Supplementary Table 6. Sensitivity analysis with COVID-19 exposure treated as a time-dependent variable. Individuals whose CVD event is before or on the day of hospitalisation are treated as non-hospitalised for covid in this analysis, while events after the day of admission are treated as hospitalised. Follow-up was considered to start at 01/03/2020 and COVID-19 exposed cases contributed to the non-exposed risk set, up to the time of their COVID-19 diagnosis. COVID-19 cases diagnosed after the censor date contributed to the models as unexposed controls. AF: atrial fibrillation; CI: confidence interval; COVID-19: coronavirus disease 2019; CVD: cardiovascular disease; HF: heart failure; HR: hazard ratio; IHD: ischaemic heart disease; MI: myocardial infarction; VTE: venous thromboembolism

Supplementary Figure 1 Flow chart of participant selection – whole cohort

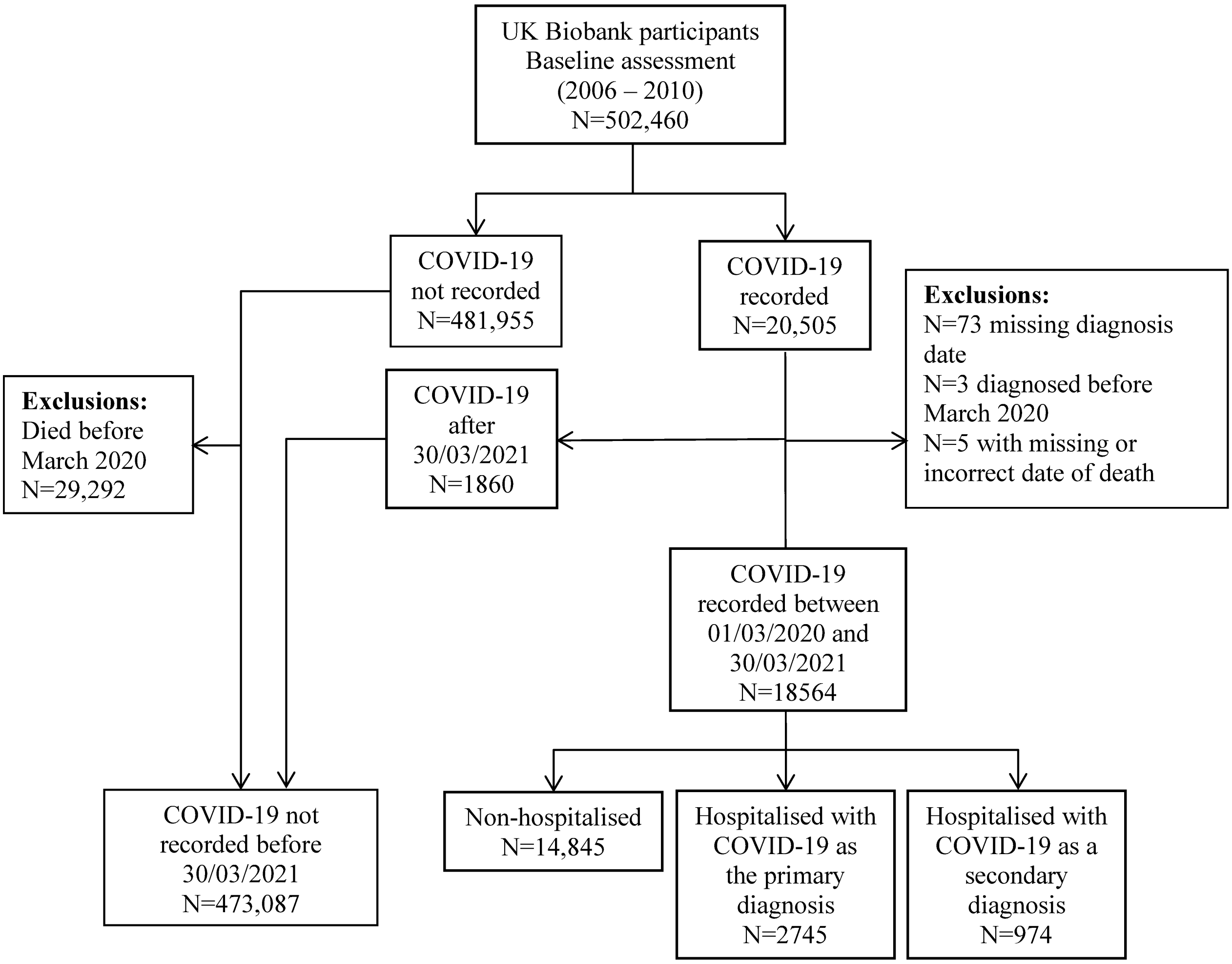

Supplement: Supplementary data [file heartjnl-2022-321492supp001.pdf]
